# Noninvasive Ultrasound Monitoring of Embryonic and Fetal Development in *Chinchilla lanigera* to Predict Gestational Age: Preliminary Evaluation of This Species as a Novel Animal Model of Human Pregnancy

**DOI:** 10.1155/2019/6319476

**Published:** 2019-05-27

**Authors:** A. Greco, M. Ragucci, R. Liuzzi, M. Prota, N. Cocchia, G. Fatone, M. Mancini, A. Brunetti, L. Meomartino

**Affiliations:** ^1^Department of Advanced Biomedical Science, University of Naples Federico II, Naples 80131, Italy; ^2^Institute of Biostructure and Bioimaging, CNR, Naples 80131, Italy; ^3^Interdepartmental Center of Veterinary Radiology, University of Naples Federico II, Naples 80137, Italy; ^4^Department of Veterinary Medicine and Animal Production, University of Naples Federico II, Naples 80137, Italy

## Abstract

Ultrasound is a noninvasive routine method that allows real-time monitoring of fetal development in utero to determine gestational age and to detect congenital anomalies and multiple pregnancies. To date, the developmental biology of *Chinchilla lanigera* has not yet been characterized. This species has been found to undergo placentation, long gestation, and fetal dimensions similar to those in humans. The aim of this study was to assess the use of high-frequency ultrasound (HFUS) and clinical ultrasound (US) to predict gestational age in chinchillas and evaluate the possibility of this species as a new animal model for the study of human pregnancy. In this study, 35 pregnant females and a total of 74 embryos and fetuses were monitored. Ultrasound examination was feasible in almost all chinchilla subjects. It was possible to monitor the chinchilla embryo with HFUS from embryonic day (E) 15 to 60 and with US from E15 to E115 due to fetus dimensions. The placenta could be visualized and measured with HFUS from E15, but not with US until E30. From E30, the heartbeat became detectable and it was possible to measure fetal biometrics. In the late stages of pregnancy, stomach, eyes, and lenses became visible. Our study demonstrated the importance of employing both techniques while monitoring embryonic and fetal development to obtain an overall and detailed view of all structures and to recognize any malformation at an early stage. Pregnancy in chinchillas can be confirmed as early as the 15^th^ day postmating, and sonographic changes and gestational age are well correlated. The quantitative measurements of fetal and placental growth performed in this study could be useful in setting up a database for comparison with human fetal ultrasounds. We speculate that, in the future, the chinchilla could be used as an animal model for the study of US in human pregnancy.

## 1. Introduction

The developmental biology of the domesticated long-tailed*Chinchilla lanigera*, a South American species, is not well characterized. Recently, it has been shown that placental and fetal metabolism and the placental vessels of chinchillas are very similar to those of humans [1, 2]. The chinchilla placenta is of the haemomonochorial labyrinthine type and therefore resembles the human villous haemomonochorial placenta, demonstrating that this species is suitable for human obstetric research and for the study of placental and fetal functions [3–5]. Females typically twin (range: 1–6 pups) after a gestation of ∼112 days (range: 105–115 days) and give birth to 2–3 litters per year, resulting in relatively fewer offspring than those of other rodent species [6]. The reproductive physiology of the hystricomorph chinchilla is different from that of myomorphic rodents typically used in biomedical research. At the same time, the chinchilla has a long gestation period and a long estrus cycle compared with other rodents; for example, the gestational period of guinea pigs ranges from 59 to 72 days, that of rats from 21 to 23 days, and that of mice from 19 to 21 days [7–10]. The long gestation and the reduced number of fetuses per gestation allow for better visualization of the embryo, longer longitudinal monitoring, and a more detailed analysis of the placenta and fetal organs. In addition, the weight of the neonatal chinchilla is relatively high (50–70 g) compared to the weight of the adult chinchilla and or of other rodent models, which facilitate instrumental ultrasonographic examination [8].

Ultrasonography is the imaging technique of choice for analyzing embryonic and fetal formation in utero [11–15]. Until a few decades ago, structural phenotyping was based on macroscopic examinations and histological techniques that only allowed the postmortem analysis of static structures. Subsequently, different imaging approaches have become available for the study of small animals, such as ultrasonography, magnetic resonance imaging (MRI), optical imaging, and confocal biomicroscopy [2, 16–18]. The ability of ultrasonography to acquire longitudinal data in real time noninvasively, the ability to analyze the morphology of nearly all the organs, and the ability to perform quantitative measurements on most of the structures have made this imaging modality particularly advantageous in the field of medical imaging. Conventional ultrasonography uses a range of frequencies, from 2 to 15 MHz, with a spatial resolution of 200–500 *μ*m; the limitations of these systems have become evident in several studies on embryonic development in the mouse, particularly during the morphogenesis phase [11]. Technological progress has also led to the development of high-frequency ultrasonography (HFUS), which achieves microscopic resolution and is thus the best imaging technique to monitor the embryonic development of small animals. HFUS systems use higher frequencies, between 40–100 MHz, and have a spatial resolution of approximately 30 *μ*m; therefore, they are useful for imaging developmental processes occurring in small organisms [11, 13].

Previously, our research group used HFUS to monitor embryonic and fetal development in mice. We assessed changes in phenotypic parameters during pregnancy and evaluated physiological fetal parameters of the principal organ development to build a database of normal structural and functional parameters of mouse development [12, 13]. Due to the chinchilla embryos' dimensions, it was not possible to monitor the entire pregnancy with HFUS (with the exception of some specific parameters), but it was necessary to use conventional ultrasound in the advanced stage of pregnancy. Up until today, there are no published reports on the use of ultrasound to monitor the fetal development of chinchillas. The aim of our research was to acquire new knowledge of the reproductive physiology of chinchillas based on the gestational similarities with humans and to assess whether this species could potentially be used in obstetric biomedical research.

## 2. Materials and Methods

The study was performed with the consent of chinchilla owners and breeders. Ethical clearance (50380-2018) from the Ethical Committee for Animal Experimentation of the University of Naples Federico II was obtained.

Healthy pregnant chinchillas were analyzed at the Interdepartmental Radiology Center of the University of Naples Federico II and at the La Plata breeding center in Cupello (CH), Italy.

We divided the pregnancy into four stages: very early stage (T1) from embryonic day (E) E15 to E30, early stage (T2) from E31 to E46, intermediate stage (T3) from E47 to E70, and advanced stage (T4) from E71 to E115. The pregnancy stage of the monitored females was determined by the date of their last birth. The chinchilla, as well as the guinea pig, begins a new estrous 12–48 h after birth [6]. From this date, we assumed a period of approximately 115 days of gestation; at parturition, both the effective time of conception and the stages of gestation were retrospectively confirmed.

### 2.1. Ultrasound Imaging

All ultrasound (US) exams were performed by an experienced veterinary ultrasonographer on awake subjects gently restrained to avoid any possible stress to the pregnant females. We monitored 35 pregnant females with a mean age of 3.5 years. Of those, 15 were in their second coupling, 10 in their first, and 10 in their third. The animals used for this study underwent a trichotomy of the pelvic and abdominal area for which no sedation was necessary since the chinchilla is a docile animal. All measurements were made by the same ultrasonographer to ensure consistency during the investigation. In each pregnant female, 2-3 embryos were imaged. The number of fetuses ultrasonographically assessed was compared to the actual number of fetuses at parturition, as communicated to us by the breeders and owners. After obtaining the US results of the entire study, the pregnancy was divided into four stages in order to longitudinally analyze the same morphometric parameter. Each chinchilla was monitored with US in a period ranging from E15 to E115, for a total of four US examinations for each chinchilla. US was performed every two weeks at the beginning of the pregnancy, at T1 and T2, and about every 40 days during T3 and T4, to avoid excessive stress on the animals. The exact day of pregnancy, in which US was performed, was retrospectively determined only after the delivery date, which was communicated to us by the breeders and the owners.

During stage T1, we used the high-frequency ultrasound system, Vevo 770 (Visualsonics, Canada), equipped with a 40 MHz high-resolution linear transducer (focal length 6 mm, depth of penetration 5–15 mm, resolution 30–40 mm axial, and 70–90 mm lateral). During the other stages, we used a US device (MyLab 30, Esaote, Firenze, IT) equipped with a 12 MHz linear probe. A series of measurements were obtained: the longitudinal and transversal diameters of the gestational sacs and the diameter and thickness of the placenta at T1. At T2, T3, and T4, we measured the size of the gestational sacs, the diameter and thickness of the placenta, and the crown-rump length (CRL). Furthermore, we measured the head diameter (HD), the body diameter (BD), the occipital-snout length (OSL), and the heart rate (HR); the femur length (FL) and the stomach were measured from T3, and eye and lens diameter were measured at T3.

### 2.2. Statistical Analysis

Continuous variables were reported as the mean ± standard deviation. Univariate analysis between each variable and embryonic day was performed using Spearman's rank correlation (Rs). Linear regression analysis was used to assess the relationships between embryonic variables and gestational time. A *p* value < 0.05 was considered statistically significant. Statistical analyses were performed using MedCalc 18.2.1 software (Ostend, Belgium).

## 3. Results

### 3.1. Ultrasonographic Findings

The ultrasonographic examination was feasible in all chinchilla subjects. Every ultrasonographic examination lasted approximately 10 min. Both techniques, HFUS and US, were able to diagnose pregnancy. However, not all fetal structures could be visualized with both techniques. In the first stage (T1), we found 74 embryos, but at the delivery, this number decreased as the 35 chinchillas effectively delivered 67 cubs. Considering the 35 pregnant females, the pregnancy loss was 11%. Since it was not possible to obtain the measurements of each parameter at every ultrasonographic examination, we reported the exact number of fetuses, for which each parameter was measured (Table 1).

It was possible to monitor the chinchilla embryos with HFUS from E15 to E60 and with US from E15 to E115. This was due to the increasing fetal dimensions that do not permit accurate measurement of several fetal structures with HFUS. The assessability of the analyzed fetal structures depended on their dimensions and thickness: the placenta was 100%, the HD was 82%, the CRL was 57%, and the femur was 26%. We summarized the assessed structures per gestational stage and the employed ultrasound technique in Table 2.

**Figure 1:**
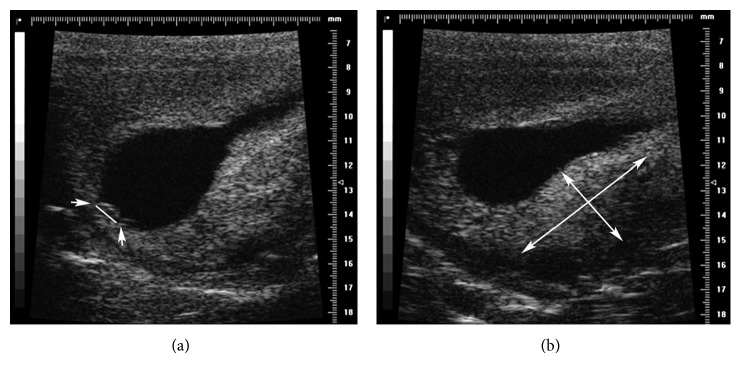
HFUS B-mode image at E15. (a) An embryo 1.06 mm in diameter (arrow) is evident. (b) The placenta (4.24 × 9.17 mm).

At T1, from E15 to E30, it was possible to visualize the gestational sac with both HFUS and US. However, the placental diameter and thickness were visualized and measured only with HFUS because the placenta at this stage is not yet discoidal in shape. In the very early stage, it was also possible to identify and measure the gestational sac and to distinguish the primitive node (Figure 1).

**Figure 2:**
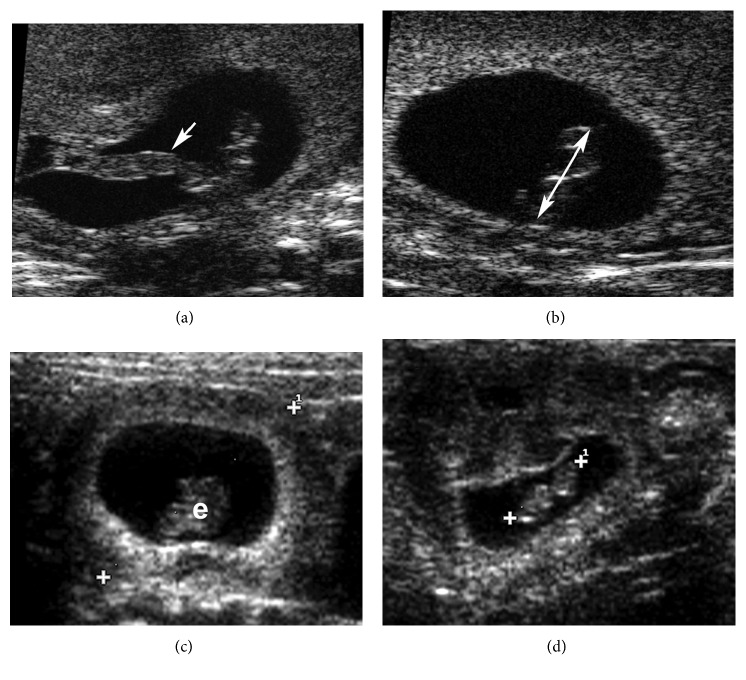
Images of a chinchilla embryo at embryonic day 31 (E31). HFUS B-mode: (a) the umbilical cord (arrow) is evident; (b) measurement of the crown-rump length (CRL) (double-headed arrow) (3.34 mm). US B-mode: (c) image of the embryo (e) and the gestational sac diameter (GSD) (16 mm); (d) measurement of the CRL (6 mm).

At T2, from E30 to E46, the placental diameter and the placental thickness were visualized with US. From E30, it was possible to measure the CRL with both HFUS and US (Figure 2), and from E38, the BD was also measurable. All these parameters were better visualized with HFUS at T2. The OSL, in particular, could be visualized and measured from E40 because, at this gestational age, the head becomes well defined.

**Figure 3:**
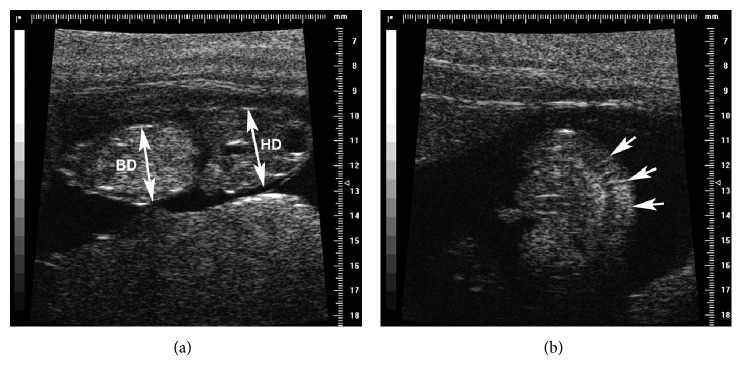
HFUS B-mode image of the embryo at embryonic day 46. (a) The head diameter (HD) (4.3 mm) and the body diameter (BD) (3.1 mm) are highlighted (arrows); (b) the neural crest (arrows) is visible.

At E30, the heartbeat was detectable for the first time with HFUS, and it was possible to visualize the completed neural tube at E46 (Figure 3).

**Figure 4:**
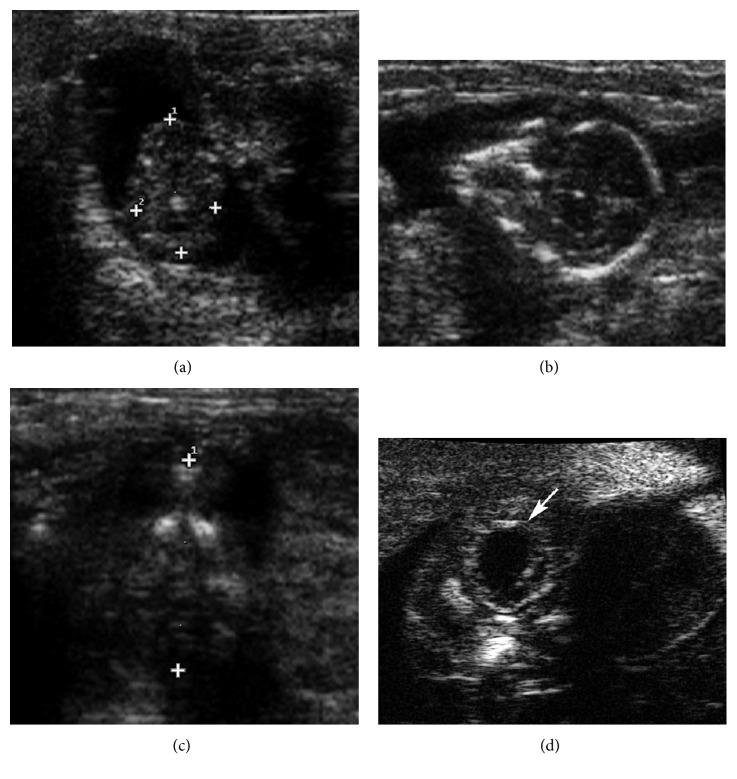
Embryonic days 49–60. US B-mode: (a) images of the head at embryonic day 49 with the measurements of the head diameter (HD) (short axis) and occipital to snout length (OSL) (long axis) (9.1 × 5.3 mm); (b) head US aspect at embryonic day 49: skull bones are hyperechoic but still not mineralized; (c) measurement of the occipital to snout length (OSL) at embryonic day 67 (12.6 mm); HFUS B-mode: (d) the eyes (arrow) and the ears are visible at E60.

From E55, it was possible to detect the stomach, the eyes, and the lenses (Figure 4).

**Figure 5:**
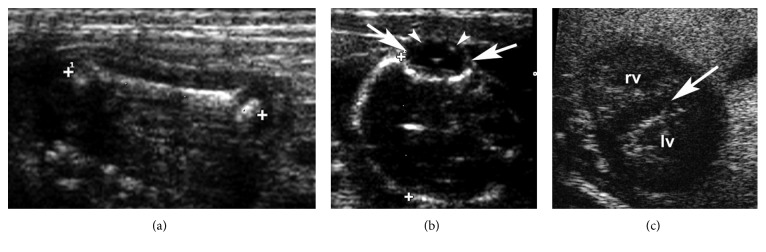
Embryonic days 98–102. US B-mode and HFUS: (a) measurement of the femur (16.5 mm); (b) measurement of the eye (arrows) and lens (arrowheads); (c) HFUS B-mode image of the heart (rv: right ventriculum; lv: left ventriculum) at embryonic day 102. The interventricular septum (arrow) is visible.

At E66, the femur became clearly visible and measurable because of increased mineralization (Figure 5(a)). In the advanced pregnancy stage T4 (E75–E115), it was no longer possible to measure the CRL due to fetal dimensions, bone mineralization was pronounced, and the dimensions of eyes and lenses were approximately the same as those in newborns (Figure 5).

From the beginning of the intermediate stage T3 (E47–E70), it was possible to monitor the chinchillas only with US due to the growing dimensions of the fetal structures. However, we were able to use HFUS to measure the anteroposterior and laterolateral lens diameters until E115. HFUS in this stage of gestation (E60) was also useful to visualize the interventricular septum of the heart and to quantify the HR until the end of pregnancy.

### 3.2. Statistical Analysis

**Figure 6:**
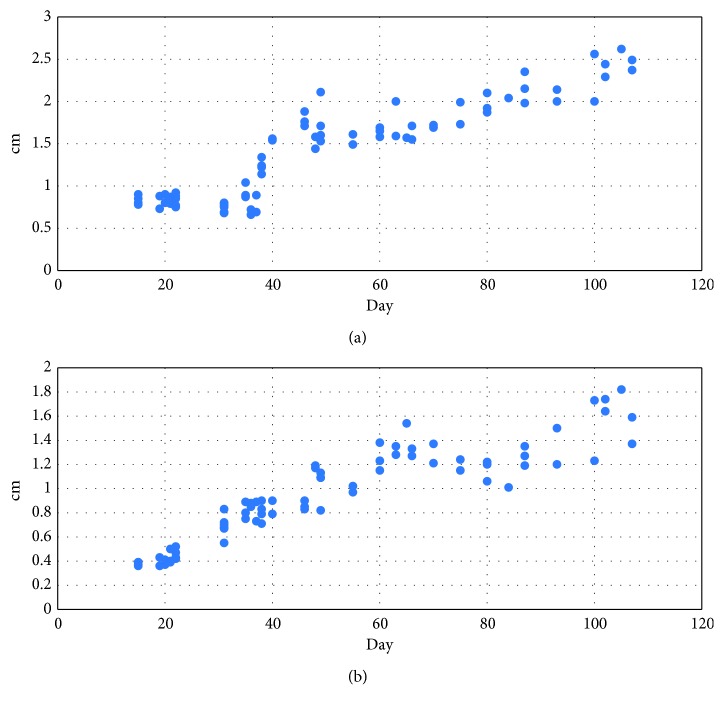
Plot of the US measurement of the placental diameter (a) and of the placental thickness (b) obtained from E15 to E115.

Summarizing the most important results of the first two stages (T1 and T2), the diameter of the placenta increases from 0.8 cm to 1.6 cm (about 2-fold) and the thickness of the placenta increases from 0.4 to 1.21 cm (about 3-fold). A plot of the analysis of the diameter and the thickness of the placenta, measured longitudinally in the four stages, is presented in Figure 6.

The longitudinal size of the gestational sacs increased from 1.17 to 2.39 cm (2-fold) until E49. Also, the CRL, HD, and BD gradually increased (CRL: 0.7 to 2.7, about 4-fold; HD: 0.25 to 0.65, about 5-fold; BD: 0.85 to 1.9 cm, about 2-fold) till the end of the pregnancy (Figures 2 and 3). The precise measurements of the parameters from the first day of pregnancy to the last are reported in Table 3.

All structural measurements, except for femoral length, were significantly correlated with gestational age. The results of correlation analysis are reported in Table 4.

Based on the period during which it was possible to measure the aforementioned variables simultaneously, they embryos measured were placed in three groups for the regression analysis: group 1 included variables measurable between time T1 to T3 (i.e.,gestational sac diameter and placental diameter and thickness); group 2 included variables measurable between time T2 to T3 (i.e.,placental diameter and thickness, CRL, HD, BD, OSL, and HR); group 3 included variables measurable between time T2 to T4 (i.e.,placental diameter and thickness, BD, OSL, HD, and HR). Using this analysis, three models for predicting gestational age were obtained (Table 5).

## 4. Discussion

We examined embryo and fetus development in the *C. lanigera* using an ultrasonographic technique, and we predicted gestational age with the analysis of ultrasound parameters.

Ultrasonography is a noninvasive method that provides a longitudinal, real time, and detailed morphological evaluation of fetuses in vivo. Furthermore, US is quick and less expensive than other imaging modalities when screening for neonatal defects. We performed a longitudinal, qualitative, and morphometric evaluation of chinchilla embryonic parameters from E15 to the last day of pregnancy using HFUS and US. As in human obstetrics, we analyzed different morphometric parameters useful to predict the gestational age of the fetus (such as the CRL or the HR) and others like the HD, the OSL, or the BD, embryo anatomical size measurements, position of the placenta, and structures, which are useful for predicting anomalies in the unborn child. The ultrasonographic exam was feasible in all subjects, even when subjects were awake. The main difficulty encountered when performing the US exam was related to the anatomy of the chinchilla, which has a highly developed caecum that can sometimes hinder the sight of some structures of interest. Furthermore, the fetuses of chinchillas have a big head that, especially in the last part of pregnancy, when mineralization is advanced, can obstruct the visualization of other anatomical structures of the fetus. Lastly, US is a subjective technique in which the expertise and capabilities of the operator are of paramount importance. In further studies, it would be useful to compare US evaluation performed by two or more operators to assess reliability, sensitivity, and specificity of the method.

Since the animals examined in this study were bred or owned as pets, we had to perform our exams on awake subjects. This circumstance and the abovementioned anatomic characteristics of the chinchilla limited the analysis of some anatomical structures. However, the morphometric data we obtained for the different parameters during pregnancy were consistent and reproducible within the same gestational stage, and no female showed any signs of stress during or following US examinations. All subjects completed their pregnancies although some fetuses were reabsorbed. Studies performed in mice under general anesthesia are more detailed; however, the use of anesthetics often affects the newborn offspring, thus disrupting the possible comparison with human ultrasound findings [19].

Our intent was to protect the well-being of the cubs and fetuses and to demonstrate the noninvasiveness and feasibility of ultrasonographic examination, which can also be performed on awake subjects. In our study, some observation days were missed because we performed only four US examinations per chinchilla (from E15 to E115). This was done to avoid excessive stress on the pregnant females. Also limiting in our study is that, in the 115 days, we performed the first two US analyses in the first 115 days, about every 2 weeks and then about every 40 days thereafter, so we lucked some temporal points of observation. This was due to difficulties reaching the breeding center and also because the chinchillas used for the study were domesticated animals and not experimental animals. Thus, we adjusted our research to suit the availability of the breeding center and owners.

We used a best-fit regression coefficient to analyze which parameters could be predicted for each gestational age. Based on our results, the placental thickness at T1–T3, the HR, and the CRL at T2-T3, and the HD and HR at T2–T4 could be used to predict the exact gestational age of the chinchilla fetuses. However, some parameters, such as the femoral length, which are used to predict gestational age in human pregnancy, have to be further evaluated to increase casuistry. Furthermore, other parameters will likely be needed to better predict gestational age. For this reason, it is useful to have a precise database of physiological morphometric parameters of embryo and fetal development during chinchilla pregnancy, even if divided in stages and not in days, in order to diagnose pathological conditions that could occur.

Since 1947, the efficiency of animal models for the study of human pathologies of pregnancy has been investigated. Even today an adequate model for the study of human pregnancy has yet to be found [20]. A recent study shows that, in animal perinatology, the use of animal models does not give adequate mathematical correlations with human pregnancy preterm birth [21]. The choice of the right animal model in human obstetrics is challenging because there are several aspects to consider. The duration of gestation, the number of fetuses for each pregnancy, and the placental morphology are all important parameters to evaluate when comparing placentation and fetal morphology between rodents and humans. Other aspects that should not be underestimated are the management costs of the animal, the ease of animal housing, and the legislation (Directive 2010/63/EU) that regulates the use of one species instead of another as an animal model for biomedical research.

The chinchilla might be more suitable as an animal model for human obstetrics than the guinea pig and other rodents because of the longer duration of pregnancy (105–115 days) [22–24]. The female chinchilla's placenta is comparable to the human haemomonochorial placenta, which is formed of only one layer of syncytiotrophoblasts, unlike the other rodents [2]. Furthermore, chinchilla cubs are fully developed, covered with hair, have already developed sight and hearing, and are able to move a few hours after birth. These characteristics make chinchilla development resemble humans, who have a longer intrauterine development than myomorphic rodents and rabbits. Finally, the reduced number of offspring (1–6, on average 1 or 2 cubs per gestation) facilitates monitoring of the same fetus with US, avoiding errors due to the presence of numerous fetuses in a single pregnancy and allowing a higher spatial resolution and sensitivity using imaging techniques. The discordant data between the number of US examinations and the number of fetuses communicated by the owners and breeders are probably due to fetal reabsorption that often occurs in this species [25].

## 5. Conclusions

In conclusion, US lasts only a few minutes and is well tolerated in chinchillas, which have proved to be a suitable animal model for the study of human pregnancy and biomedical research in general [25]. Our research has provided important data, which allow the division of the gestation in four stages. This is particularly relevant in chinchillas since estrus is often silent and does not permit detection of the exact time of mating; with ultrasound, it is possible to establish the exact stage of pregnancy because of the reliability of morphometric measurements. One limitation of the study is the fact that we analyzed each chinchilla only four times during the entire pregnancy, which resulted in some longitudinal observations being missed. Further studies will be carried out by our group to obtain all morphometric measurements that will cover the entire pregnancy of the chinchilla.

## Figures and Tables

**Table 1:** Number of embryos for each measurement.

Type of measurement	Gestational age (days)	Number of embryos measured
Longitudinal sac diameter	E15–E49	44
Transversal sac diameter	E15–E49	44
Placental diameter	E15–E107	74 to 67
Placental thickness	E15–E107	74 to 67
Lens	E60–E107	23
Eye	E55–E107	22
Head diameter	E31–E107	61
Occipital snout length	E40–E107	61
Heart rate (BPM)	E31–E107	55
Stomach	E55–E107	33
Longitudinal body diameter	E38–E107	61
Femur	E66–E107	19
Crown-rump length	E31–E70	42

E: embryonic day; BPM: beats per minute.

**Table 2:** Ultrasound visible fetal anatomic structure per gestational stage.

Stage	Anatomic embryo/fetal structure	Ultrasound techniques
T1 (E15–E30)	Longitudinal sac diameter	HFUS, US
	Transversal sac diameter	HFUS, US
	Placental diameter	HFUS
	Placental thickness	HFUS

T2 (E31–E46)	Longitudinal sac diameter	HFUS, US
	Transversal sac diameter	HFUS, US
	Placental diameter	HFUS, US
	Placental thickness	HFUS, US
	Head diameter	HFUS, US
	Occipital snout length	HFUS, US
	Heart rate (BPM)	HFUS, US
	Longitudinal body diameter	HFUS, US
	Crown-rump length	HFUS, US

T3 (E47–E70)	Placental diameter	HFUS, US
	Placental thickness	HFUS, US
	Lens	HFUS, US
	Eye	HFUS, US
	Head diameter	HFUS, US
	Occipital snout length	HFUS, US
	Heart rate (BPM)	HFUS, US
	Stomach	HFUS, US
	Longitudinal body diameter	HFUS, US
	Femur	HFUS, US
	Crown-rump length	US

T4 (E71–E115)	Placental diameter	US
	Placental thickness	US
	Lens	US
	Eye	US
	Head diameter	US
	Occipital snout length	US
	Heart rate (BPM)	US
	Stomach	US
	Longitudinal body diameter	US
	Femur	US
	Crown-rump length	US

BPM: beats per minute.

**Table 3:** Morphometric evaluation (in mm).

Type of measurement	Measurement at the beginning of pregnancy (mean ± SD)	Measurement at the end of pregnancy (mean ± SD)
Longitudinal sac diameter	11.7 ± 1.6 (E15)	23.9 ± 2.9 (E49)
Transversal sac diameter	8.9 ± 2.0 (E15)	18.6 ± 1.8 (E49)
Placental diameter	8.3 ± 0.6 (E15)	20.5 ± 5.6 (E107)
Placental thickness	4.1 ± 0.5 (E15)	13.6 ± 2.4 (E107)
Lens diameter	1.4 ± 0.3 (E60)	2.2 ± 0.8 (E107)
Eye axis	3.2 ± 0.6 (E55)	4.9 ± 1.2 (E107)
Head diameter	2.6 ± 0.7 (E31)	16.4 ± 1.7 (E107)
Occipital to snout length	5.0 ± 0.4 (E40)	26.9 ± 5.2 (E107)
Heart rate (BPM)	154 ± 13.8 (E31)	138 ± 39 (E107)
Stomach diameter	3.7 ± 1.0 (E55)	7.3 ± 2.1 (E107)
Body diameter (long axis)	8.5 ± 2.2 (E38)	19.0 ± 4.3 (E107)
Femur length	9.4 ± 0.7 (E66)	14.5 ± 4.3 (E107)
Crown-rump length	7.1 ± 3.3 (E31)	27.0 ± 7.8 (E70)

BPM: beats per minute.

**Table 4:** Spearman's correlation coefficient and significance of each embryonic parameter.

Embryonic structure	Correlation coefficient	*p*
Longitudinal sac diameter	0.765	<0.0001
Transversal sac diameter	0.683	<0.0001
Placental diameter	0.841	<0.0001
Placental thickness	0.902	<0.0001
Lens diameter	0.639	0.0014
Eye axis	0.313	0.1914
Head diameter	0.942	<0.0001
Occipital to snout length	0.923	<0.0001
Heart rate (BPM)	−0.362	0.0062
Stomach diameter	0.517	0.0114
Body diameter (long axis)	0.919	<0.0001
Crown-rump length	0.71 ± 0.33 (E31)	2.70 ± 0.78 (E70)

BPM: beats per minute.

**Table 5:** Best-fit regression coefficient and standard error for the three groups of variables.

Gestation time	Model	Set of parameters	Coefficient	Standard error	*p*
T1–T3	1	Placental thickness	2.54	0.15	<0.0001
		Constant	0.007		
			

T2-T3	2	Heart rate in beats per minute	−0.009	0.001	<0.0001
		Crown-rump length	0.30	0.03	<0.0001
		Constant	3.19		

T2–T4	3	Head diameter	1.26	0.06	<0.0001
		Heart rate in beats per minute	−0.004	0.001	0.0008
		Constant	2.34		

Model 1 uses placental thickness to predict the gestational age from T1 to T3. Model 2 uses HR and CRL to predict gestational age from T2 to T3. Finally, model 3 uses HD and HR to predict gestational age from T2 to T4.

## Data Availability

The ultrasound data used to support the findings of this study are included within the article.

## References

[1] Johnsen S. L., Wilsgaard T., Rasmussen S., Sollien R., Kiserud T. (2006). Longitudinal reference charts for growth of the fetal head, abdomen and femur. *European Journal of Obstetrics & Gynecology and Reproductive Biology*.

[2] Mikkelsen E., Lauridsen H., Nielsen P. R. (2016). The chinchilla as novel animal model of pregnancy. *Royal Society Open Science*.

[3] Grigsby P. (2016). Animal models to study placental development and function throughout normal and dysfunctional human pregnancy. *Seminars in Reproductive Medicine*.

[4] King B. F., Tibbitts F. D. (1976). The fine structure of the chinchilla placenta. *American Journal of Anatomy*.

[5] Tibbitts F. D., Hillemann H. H. (1959). The development and histology of the chinchilla placentae. *Journal of Morphology*.

[6] Mastromonaco G. F., Cantarelli V. I., Galeano M. G., Bourguignon N. S., Gilman C., Ponzio M. F. (2015). Non-invasive endocrine monitoring of ovarian and adrenal activity in chinchilla (*Chinchilla lanigera*) females during pregnancy, parturition and early post-partum period. *General and Comparative Endocrinology*.

[7] Bonney E. A. (2013). Demystifying animal models of adverse pregnancy outcome: touching bench and bedside. *American Journal of Reproductive Immunology*.

[8] Georgiades P., Ferguson-Smith A. C., Burton G. J. (2002). Comparative developmental anatomy of the murine and human definitive placentae. *Placenta*.

[9] Soares M. J., Chakraborty D., Karim Rumi M. A., Konno T., Renaud S. J. (2012). Rat placentation: an experimental model for investigating the hemochorial maternal-fetal interface. *Placenta*.

[10] Friesen-Waldner L. J., Sinclair K. J., Wade T. P. (2015). Hyperpolarized [1-13C] pyruvate MRI for noninvasive examination of placental metabolism and nutrient transport: a feasibility study in pregnant Guinea pigs. *Journal of Magnetic Resonance Imaging*.

[11] Shung K. K. (2009). High frequency ultrasonic imaging. *Journal of Medical Ultrasound*.

[12] Chang C.-P., Chen L., Crabtree G. R. (2003). Sonographic staging of the developmental status of mouse embryos in utero. *Genesis*.

[13] Greco A., Ragucci M., Coda A. R. D. (2013). High frequency ultrasound for in vivo pregnancy diagnosis and staging of placental and fetal development in mice. *PLos One*.

[14] Greco A., Coda A. R. D., Albanese S. (2015). High-frequency ultrasound for the study of early mouse embryonic cardiovascular system. *Reproductive Sciences*.

[15] Marinac-Dabic D., Krulewitch C. J., Moore R. M. (2002). The safety of prenatal ultrasound exposure in human studies. *Epidemiology*.

[16] Zhou Y. Q., Foster F. S., Qu D. W., Zhang M., Harasiewicz K. A., Adamson S. L. (2002). Applications for multifrequency ultrasound biomicroscopy in mice from implantation to adulthood. *Physiological Genomics*.

[17] Mu J., Slevin J. C., Qu D. (2008). In vivo quantification of embryonic and placental growth during gestation in mice using micro-ultrasound. *Reproductive Biology and Endocrinology*.

[18] Turnbull D. H., Bloomfield T. S., Baldwin H. S., Foster F. S., Joyner A. L. (1995). Ultrasound backscatter microscope analysis of early mouse embryonic brain development. *Proceedings of the National Academy of Sciences*.

[19] Lee S., Chung W., Park H. (2017). Single and multiple sevoflurane exposures during pregnancy and offspring behavior in mice. *Pediatric Anesthesia*.

[20] Miller H. C. (1947). The effect of pregnancy complicated by alloxan diabetes on the fetuses of dogs, rabbits and rats. *Endocrinology*.

[21] Nielsen B. W., Bonney E. A., Pearce B. D., Donahue L. R., Sarkar I. N., For the Preterm Birth International Collaborative (PREBIC) (2016). A cross-species analysis of animal models for the investigation of preterm birth mechanisms. *Reproductive Sciences*.

[22] Jiménez J. E. (1996). The extirpation and current status of wild chinchillas *Chinchilla lanigera* and c. brevicaudata. *Biological Conservation*.

[23] Busso J. M., Ponzio M. F., De Cuneo M. F. (2007). Non-invasive monitoring of ovarian endocrine activity in the chinchilla (*Chinchilla lanigera*). *General and Comparative Endocrinology*.

[24] Bekyürek T., Liman N., Bayram G. (2002). Diagnosis of sexual cycle by means of vaginal smear method in the chinchilla (*Chinchilla lanigera*). *Laboratory Animals*.

[25] Andersen M. D., Alstrup A. K. O., Sondergaard C. (2018). *Animal Model of Fetal Medicine and Obstetrics in Intechopen*.

